# The Olfactory System of Zebrafish as a Model for the Study of Neurotoxicity and Injury: Implications for Neuroplasticity and Disease

**DOI:** 10.3390/ijms20071639

**Published:** 2019-04-02

**Authors:** Erika Calvo-Ochoa, Christine A. Byrd-Jacobs

**Affiliations:** Department of Biological Sciences, Western Michigan University, Kalamazoo, MI 49008-5410, USA; erika.calvoochoa@wmich.edu

**Keywords:** zebrafish, olfactory system, olfactory organ, olfactory bulb, toxicant, neuroplasticity, regeneration, injury

## Abstract

The olfactory system, composed of the olfactory organs and the olfactory bulb, allows organisms to interact with their environment and through the detection of odor signals. Olfaction mediates behaviors pivotal for survival, such as feeding, mating, social behavior, and danger assessment. The olfactory organs are directly exposed to the milieu, and thus are particularly vulnerable to damage by environmental pollutants and toxicants, such as heavy metals, pesticides, and surfactants, among others. Given the widespread occurrence of olfactory toxicants, there is a pressing need to understand the effects of these harmful compounds on olfactory function. Zebrafish (*Danio rerio*) is a valuable model for studying human physiology, disease, and toxicity. Additionally, the anatomical components of the zebrafish olfactory system are similar to those of other vertebrates, and they present a remarkable degree of regeneration and neuroplasticity, making it an ideal model for the study of regeneration, reorganization and repair mechanisms following olfactory toxicant exposure. In this review, we focus on (1) the anatomical, morphological, and functional organization of the olfactory system of zebrafish; (2) the adverse effects of olfactory toxicants and injury to the olfactory organ; and (3) remodeling and repair neuroplasticity mechanisms following injury and degeneration by olfactory toxicant exposure.

## 1. Introduction

The olfactory system plays a pivotal role in animal survival since it detects and discriminates myriad odor signals and mediates important behaviors such as foraging, mating, social behavior, and assessing danger [1,2]. The swift modification of odor-mediated behaviors as a result of a changing environment requires the olfactory system to be very dynamic; as a result, the olfactory system presents widespread plasticity mechanisms to adjust continuously in response to odors [3,4,5]. In addition, peripheral olfactory organs are particularly vulnerable to direct exposure to pollutants, heavy metals, chemical neurotoxicants, infectious agents, and injury, making olfactory plasticity crucial [6].

It is well established that the functional and structural organization of the olfactory system, as well as olfactory transduction mechanisms, have remained evolutionary conserved in vertebrates [7,8,9,10,11,12,13]. Among these animals, the zebrafish (*Danio rerio*), a small freshwater teleost fish, has emerged as a valuable and relevant model for studying human physiology, disease, and toxicity. The major organs and tissues of zebrafish share molecular, anatomical, and physiological features with their mammalian counterparts, and it has been established that over 70% of zebrafish genes are shared with humans [14,15,16]. Additionally, zebrafish is notable for various experimental and husbandry advantages when compared to mammalian models, including faster development, higher fertility, lower maintenance costs, and easier genetic manipulation and screening, among many others.

In particular, zebrafish is an ideal model for studying the olfactory system, since its anatomical components are more accessible in this animal than other vertebrates. This makes the zebrafish olfactory system very amenable to experimental manipulations with less invasive techniques, increasing the rate of survival and recovery [10,11,17,18,19]. Another key characteristic of zebrafish is that it presents a high degree of central nervous system (CNS) regeneration, including the olfactory system, following damage [18,20,21,22,23,24,25]. This feature makes the olfactory system of zebrafish an ideal model for the study of regeneration, reorganization, and repair mechanisms following toxicant exposure or direct injury.

Here, we will provide a comprehensive overview of (1) the anatomical, morphological, and functional organization of the olfactory system of zebrafish; (2) the adverse effects of neurotoxicants, chemical damage, and physical injury in the olfactory organ and olfactory bulb; and (3) remodeling and repair neuroplasticity mechanisms following toxicant exposure and injury to the olfactory organ. This review will serve as a foundation for the use of zebrafish as a useful and robust model for assessing the effects of olfactory toxicants and injury.

## 2. The Olfactory System of Zebrafish: Anatomical, Morphological, and Functional Organization

### 2.1. The General Organization of the Olfactory System of Zebrafish

**Figure 1:**
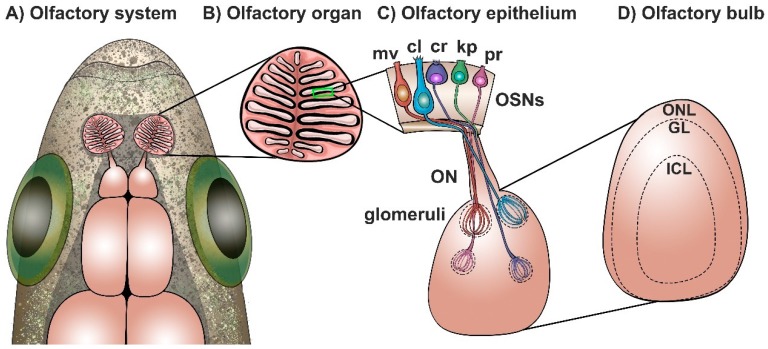
Anatomical and morphological organization of the zebrafish olfactory system. (**A**) Localization of the olfactory system in zebrafish. Dorsal side is shown; rostral side is located upwards; (**B**) Olfactory organ morphology. Olfactory sensory epithelium arranged in lamellae is shown in black; (**C**) Olfactory epithelium (OE), composed of the following olfactory sensory neurons (OSNs): microvillous (mv); ciliated (cl); crypt (cr); kappe (kp); and pear (pr) OSNs. OSNs extend their axons to the olfactory bulb via the olfactory nerve (ON) to form discrete glomeruli; (**D**) Olfactory bulb organization in three laminae: olfactory nerve layer (ONL); glomerular layer (GL); and intracellular layer (ICL).

The overall architecture and functional organization of the olfactory system of zebrafish is analogous to that of other vertebrates and has been described in detail. It is formed by two main structures: a pair of peripheral olfactory organs, or rosettes, located in the nasal cavity connected to the olfactory bulbs, which constitute the rostral-most forebrain region (Figure 1A) [9,10,17,26,27,28]. The olfactory organ is constituted primarily by a sensory epithelium consisting of olfactory sensory neurons (OSNs) that respond to odor molecules, or odorants. OSNs extend axonal projections to the olfactory bulbs, forming a fasciculated connecting fiber known as the olfactory nerve [7,29]. OSN axons reaching the olfactory bulbs form discrete structures known as glomeruli, where they form synapses with bulbar mitral cells [30,31,32,33]. These neurons extend axons to the forebrain in bundles known as olfactory tracts, where they relay signals to telencephalic olfactory-processing areas: the posterior zone of the dorsal telencephalon (Dp); the ventral nucleus of the central telencephalon (Vv); the posterior tuberculum (PT); and the right habenula (rHb). Olfactory information is processed and decoded in these telencephalic centers in order to elicit odorant-mediated behaviors [34,35,36,37]. In addition to having a well-characterized morphology and neuronal circuitry, the olfactory system of zebrafish presents remarkable regeneration, repair, and reorganization mechanisms in basal states in response to injury. Both the olfactory organ and the olfactory bulb present continuous neurogenesis and neuronal turnover throughout the organism’s lifespan [24,38,39,40,41,42]. These features make the olfactory system of zebrafish an ideal model to study mechanisms of olfaction processing, olfactory dysfunction, and regeneration following damage.

### 2.2. The Olfactory Organ of Zebrafish

The olfactory organ of zebrafish includes epithelium arranged in several lamellae that converge in a central non-sensory raphe, forming a bilaterally symmetrical, cup-shaped structure known as the rosette. Lamellae are composed of a continuous sensory area, found in the central and medial region of the rosettes, as well as a surrounding non-sensory epithelium located dorsally (Figure 1B) [10,43,44]. The rosette’s sensory region is a characteristic pseudostratified columnar epithelium formed primarily by olfactory sensory neurons (OSNs), as well as basal and supporting cells [43]. OSNs are bipolar sensory neurons that span from the basal lamina to the apical region of the epithelium, where they detect water-borne odorants. These neurons project their axons through the basal lamina, where they bundle within the lamina propia and project to the nearby olfactory bulbs, forming the olfactory nerve. There are five types of OSNs described in zebrafish: ciliated (cl); microvillous (mv); crypt (cr); and the recently described kappe (kp) and pear (pr) neurons (Figure 1C). These OSNs exhibit different morphologies, molecular markers, and profiles and are differentially located throughout the epithelial layer: ciliated OSN somata are found basally and have an elongated morphology with a long apical dendrite containing cilia; microvillous neuron somata can be located at intermediate depths of the epithelium with apical microvilli stemming from a thick dendrite; crypt neurons are found apically and present a spherical, short cell body with some cilia and microvilli; kappe OSNs are found on the apical side of the olfactory epithelium (OE) and have a short and globose shape characterized by an apical microvilli-bearing cap; and pear OSNs, which are also located apically, present a pear-shaped morphology as well as very short apical dendrites [43,45,46,47,48,49,50].

OSNs express a combination of discrete olfactory receptors (ORs), and groups of OSNs expressing the same olfactory receptors are positioned in defined zones within the rosette [38,51,52]. Evidence suggests that zebrafish OSNs follow the “one neuron–one receptor” configuration that has been described in mammals, although a couple of exceptions in ciliated OSNs have been reported [44,53,54]. Furthermore, axons of OSNs expressing the same ORs, regardless of their location in the olfactory epithelium, converge to a single glomerulus in the olfactory bulb (Figure 1C) [31,50,54,55,56,57,58].

ORs belong to the G protein-coupled receptor (GPCR) superfamily and are further divided in three types: (1) olfactory receptors (ORs), the most abundant type, further divide into families and subfamilies [27,38,44,59,60]; (2) trace amine-associated receptors (TAARs) detect volatile amines [61,62,63,64,65,66]; and (3) vomeronasal receptors (V1Rs, V2Rs) are described as putative pheromone receptors [65,67,68,69,70]. Ciliated OSNs, which preferentially respond to bile acids, express OR-type and TAAR-type receptors coupled to Gαolf [56,61,71]. Microvillous OSNs express Gαi-coupled V1Rs, which are activated by amino acids [65,72,73]. Moreover, based on epithelial location and the expression of different OSN molecular markers, it has been suggested that microvillous OSNs also express V2R, possibly through Gαo signaling [70,73,74,75]. On the other hand, crypt neurons express a single V1R-type receptor, V1R4, associated with Gαi, and although their ligands are unknown, it has been suggested that these receptors respond to pheromones [45,47,49]. Kappe neurons are immunoreactive to Gαo but their cognate receptors are still unknown [46]. Finally, the recently discovered pear OSNs express a novel receptor A2c, responsive to adenosine and adenine nucleotides [50]. Interestingly, crypt, kappe, and pear OSNs each project to a single, identifiable glomerulus in the olfactory bulb. Crypt and kappe OSNs project to two distinct glomeruli in the mediodorsal cluster, mdG2 and mdG5, respectively, whereas pear OSNs project to lG2 located in the lateral cluster [31,45,46,50]. Olfactory receptors mediate the transduction from odorant chemical signals to odor-evoked electrical signals through the regulation of cAMP. This second messenger regulates the opening of cation channels, with the ensuing opening of calcium-gated chloride channels, which in turn produce odor-evoked currents that are transmitted to the olfactory bulb [76,77].

In sum, the anatomical and functional organization of the olfactory organ, with a defined topographic map of OSNs and their cognate olfactory receptors, creates chemotopical domains of olfactory processing that enhance the discrimination of thousands of individual odorants and their combination in a precise fashion.

### 2.3. The Olfactory Bulb of Zebrafish

The olfactory bulb (OB) is a paired brain structure located in the most rostral region of the forebrain, connected to the olfactory organ by a short olfactory nerve; it constitutes the central relay of the olfactory system where olfactory information is processed and transmitted to telencephalic areas. The morphological organization and circuitry of the zebrafish OB are well understood and simpler than in mammals. The OB is organized in three diffuse laminae: (1) the olfactory nerve layer (ONL), formed by OSN axons [10,54]; (2) the glomerular layer (GL), consisting of approximately 140 spherical glomeruli—these structures are the functional and anatomical units for odor processing, formed by olfactory axons terminals and apical dendrites from mitral and tufted cells, ruffed cells, glia, and periglomerular cell interneurons [10,31,33,78]; and (3) the internal cell layer (ICL), consisting of mitral and tufted projection neuron somata, as well as granule cell dendrites [33,78,79,80] (Figure 1D).

The OB receives sensory input from peripheral OSN axons that segregate to form discrete glomeruli. About 140 glomeruli have been identified, with 27 glomeruli being easily recognized. These are organized in nine clusters based on location, OR expression, and size and are designated as follows: dorsal glomerular (dG), dorso-lateral (dlG), lateral (lG), medio-dorsal (mdG), medio-anterior (maG), medio-posterior (mpG), ventro-anterior (vaG), ventro-posterior (vpG), and ventro-medial (vmG) [17,31]. Axons of OSNs that express the same ORs, and thus respond to the same subset of odorants, converge on an individual ipsilateral glomerulus where they form glutamatergic synapses with the mitral/tufted projection neurons. Additionally, periglomerular and granular interneurons mediate feedback inhibition and modulate the activity of mitral cells [31,32,33,55,56,57,81]. Mitral and tufted cells relay output signals to olfactory-processing regions in the telencephalon and the habenular nuclei in parallel axonal bundles that form the medial (MOT) and lateral olfactory tracts (LOT) [10,31,35].

It is well established that the glomerular topographical organization of the OB creates a spatial representation of olfactory input from the OSNs that is conserved between both bulbs. Ciliated and microvillous OSNs project to specific glomeruli in a mutually exclusive manner; therefore, no glomerulus is innervated by both types of OSNs (Figure 1C). Ciliated sensory neurons, responsive to bile salts, project axons to dorsal, ventral, and medial glomeruli, whereas the amino acid-responsive microvillous OSNs project mostly to lateral glomeruli. Crypt, kappe, and pear OSNs innervate three discrete glomeruli: mdG2, mdG5, and lG2, respectively [31,35,45,50,56]. This precise glomerular organization leads to two main pathways of odor information processing: the MOT processes information from pheromones and bile salts, mediating reproductive and alarm behavior, whereas the LOT processes feeding information and behavior in response to amino acids [2,34,35,37,57,82,83].

To summarize, the topographical map of OSNs expressing similar types of ORs in the olfactory sensory epithelium, followed by the clustered axonal projection of these OSNs in specifically located glomeruli, elicits the representation of different odorant categories in the olfactory bulb. This odor map allows glomeruli to process and enhance responses to specific odorants with exquisite precision. Moreover, the further organized odor-processing output pathways to higher brain centers permit eliciting of specific odor-mediated behavioral responses crucial for zebrafish survival, such as foraging, mating, and social behaviors.

## 3. Assessing Olfaction and Olfactory Impairment in Zebrafish by Odor-Mediated Behavioral Tasks

In a fast-changing environment, olfaction provides information regarding environmental conditions such as food availability, kinship, mating availability, danger signals, and death, among many others. Fish rely on these olfactory signals to rapidly adjust their behaviors in order to survive. Odors mediate pivotal survival behaviors in fish, including feeding, alarm, schooling, avoidance response, mating, and migration. Modification of these behaviors requires that the olfactory system responds to olfactory cues in a prompt and dynamic fashion. Zebrafish offers a favorable model for studying both the mechanisms that underlie odor-sensing and olfactory alterations, as well as their behavioral effects. Regardless of the molecular mechanism behind them, olfactory alterations can be functionally grouped into three categories: (1) anosmia, a complete loss of olfaction; (2) hyposmia, a reduced olfaction capacity; and (3) dysosmia, dysregulated olfactory processing [6]. These alterations in olfaction can be assessed by a variety of odor discrimination tasks based on distinguishable and quantifiable behaviors that have been widely described in the literature [3,84,85,86,87,88,89].

Zebrafish initiate easily identifiable swimming patterns in response to diverse olfactory cues. General chemotaxis behavior includes attraction towards positive chemical cues (e.g., food) and avoidance to aversive chemical signals (e.g., predators). Chemically mediated behaviors are characterized by increased swimming speeds as fish follow or avoid the odorant source [86,89]. When exposed to food-associated odorants (i.e., amino acids, and possibly nucleotides), zebrafish exhibit appetitive olfactory behaviors, such as an increased rate of swimming and distance traveled with frequent turns (>90°) in order to orient themselves to the source of the odorant [84,86,90,91,92,93]. On the other hand, pheromones and bile salts promote social-related behaviors in zebrafish. Bile salts allow zebrafish to recognize its kin, reflected by exhibiting attraction towards familiar fish, and also promote schooling behaviors in which familiar fish form groups and swim together in a coordinated manner [86,94,95]. Pheromones elicit sexual behaviors such as courtship and spawning. Courtship behaviors in zebrafish are complex and necessary for promoting spawning and assuring reproductive success. During courtship, males swim towards, circle around, and attempt to touch the female in a tail–nose manner in order to entice her to spawn. Receptive females may approach, escort, or lead courting males before spawning [86,96,97,98]. On the other hand, alarm behaviors are activated by a mixture of chemical compounds released by specialized skin cells in response to damage, indicating potential injury caused by predation. These chemicals promote anti-predatory responses characterized by a fast avoidance with directional changes, freezing, and diving to the bottom of the tank [99,100,101,102].

**Figure 2:**
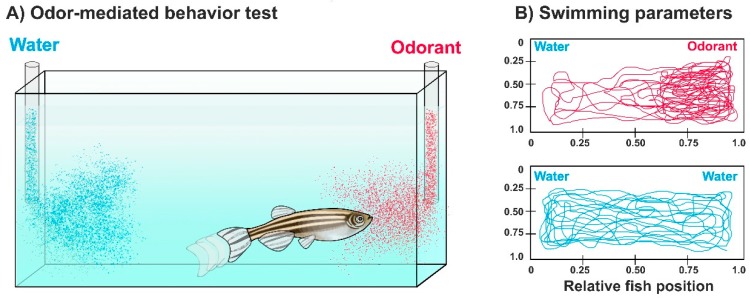
Odor-mediated behavioral tasks in zebrafish. (**A**) Odor-elicited swimming behaviors experimental setup. Individual fish are placed in either a rectangular or circular (not shown) experimental tank with two odorant delivery tubes collinearly positioned. An odorant is administered in one tube while water is simultaneously delivered in the other tube. Fish swimming patterns are recorded with a video camera (not shown); (**B**) Swimming trajectory of zebrafish after (**top**) odorant or (**bottom**) water exposure. Both swimming trajectory and time spent in each quadrant can be assessed with this test. This example depicts one of several swimming parameters that can be studied using this experimental setup.

A standardized quantitative analysis of odor-mediated behaviors of zebrafish can be accomplished by recording swimming movements of individual zebrafish before and after odorant exposure in an experimental tank, followed by manual observations or video-tracking software used for behavioral analysis (Figure 2). The latter reduces human errors and produces high-throughput and automatic quantification of swimming parameters, such as swimming trajectory, speed, distance traveled, number of turns, etc. [103,104,105]. Moreover, complex social behaviors such as mating or schooling can be automatically quantified by specialized tracking tools and 3D-video analysis software [106,107,108,109].

The increasing number of studies assessing and standardizing behavioral tasks in zebrafish has shown that these responses are robust, consistent, and comparable to other fish species and mammals, as described in the Zebrafish Behavioral Catalog [85,86,89]. In particular, odor-mediated behavioral tasks are emerging as reliable and useful tools to study functional aspects of olfaction and olfactory dysfunction in zebrafish, as well as their underlying mechanisms.

## 4. The Olfactory System of Zebrafish as a Model of Neuroplasticity (and Disease) Following Neurotoxicant Exposure and Injury

The fish olfactory sensory epithelium (OE) is in direct contact with the aquatic environment, and thus it is particularly vulnerable to exposure to waterborne pollutants, chemical toxicants, and direct injury. Damage to the olfactory system can impair olfactory function through various mechanisms affecting its different components: (1) by acting as olfactory signals (i.e., binding directly to ORs and reducing sensing of odorants) [6]; (2) by reducing odor perception and processing (e.g., through OSN injury or death) [19]; and (3) by affecting odor-dependent behaviors (e.g., reducing social behaviors) [95]. The effects of toxicants and injury on olfactory organ morphology can be studied by assessing tissue and cellular alterations through histological, immunohistochemical, and ultrastructural techniques. Furthermore, zebrafish is highly amenable to electrophysiological and optical techniques to monitor neural activity. Thus, OSNs’ response to odorants can be evaluated by their electrophysiological properties through electro-olfactography (EOG), and by in vivo calcium imaging of glomerular activity [50,58,66].

Given that odors mediate behaviors essential for survival, the olfactory system is equipped with wide-ranging neuroplasticity, remodeling, and regeneration mechanisms following damage [3,4,5,18,19,20], thus making it an excellent model to study repair responses following toxicant exposure and injury. In this section, we review results from several studies that have addressed the impact of a variety of toxicants, as well as the morphological and behavioral effects of chemical and physical lesioning on the olfactory system of zebrafish, as well as regeneration and repair mechanisms following damage. A summary of the results of these studies can be found in Table 1.

### 4.1. Effects of Heavy Metal Exposure on the Zebrafish Olfactory Epithelium

The study of the hazardous effects of heavy metals on olfaction and odorant-mediated behaviors is of particular interest, since these are widespread, neurotoxic airborne and waterborne pollutants resulting from industrial activities, nuclear energy production, mining, military activities, and fertilizer use, among other anthropogenic activities. Metal ions are particularly neurotoxic since they affect the activity of Ca^2+^ and Na^+^ channels, either by blocking the current or by modifying the channel-gating properties; these alterations modify neuronal excitability and disrupt intracellular Ca^2+^ homeostasis, which can lead to cell death [110,111]. Moreover, it has also been suggested that some ORs can function as metalloproteins or contain metal-binding sites, therefore increasing OSN susceptibility to toxic concentration of metal ions [112,113].

**Figure 3:**
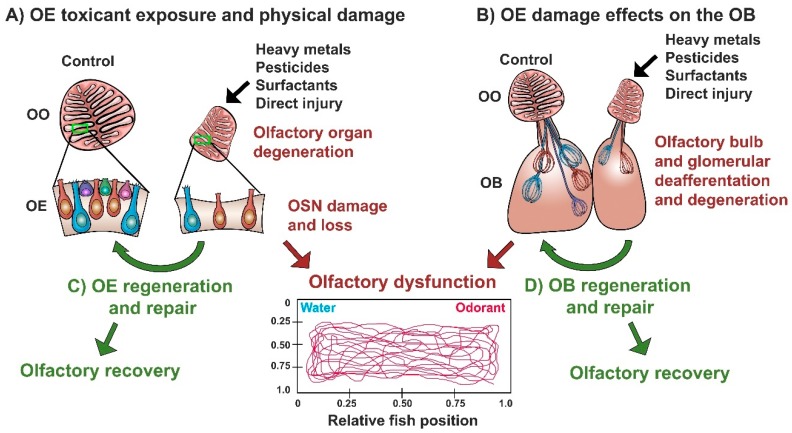
Toxicant exposure and physical lesioning effects on the olfactory epithelium and its subsequent regeneration. (**A**) Degeneration and atrophy of the olfactory organ (OO), olfactory epithelium (OE), and olfactory sensory neurons (OSNs) following exposure to some toxicants and injury paradigms, some of which lead to olfactory dysfunction; (**B**) Effects of olfactory epithelium damage due to exposure to some toxicants and direct injury on the olfactory bulb, some of which lead to olfactory dysfunction; (**C**) Olfactory epithelium regeneration and repair following damage, leading to olfactory functional recovery; (**D**) Olfactory bulb regeneration and repair following damage to the OE, leading to olfactory functional recovery.

Several groups have investigated the harmful effects of heavy metal exposure to the olfactory system of zebrafish (Figure 3A). Cadmium (Cd) exposure produces significant olfactory epithelium alterations, including reduction in OSNs and disruption in olfactory epithelium neurogenesis, through an increase in the generation of reactive oxygen species. These olfactory alterations correlate with a long-term decrease in alarm and avoidance responses [88,114,115,116,117,118,119]. Exposure to Copper (Cu) causes marked OSN death, with a preferential loss of ciliated in comparison to microvillous OSNs; impairs the transcription of a wide variety of genes involved in olfactory processing, such as ORs and ionic channels; and reduces the recognition of bile salts while impairing odor-mediated alarm responses [120,121,122,123,124]. Cobalt (Co) is a naturally occurring and essential metal at low concentrations, but it is an environmental contaminant when found in high concentrations. Chronic Co exposure in zebrafish induces acute damage to the olfactory organs through increasing oxidative stress and apoptosis. These alterations in olfactory epithelium were associated with changes in swimming and schooling behavior [125,126]. Nickel (Ni) is a potent olfactory toxicant and widespread airborne and waterborne contaminant. Ni exposure induces anatomical disturbances in the olfactory epithelium and a reduction of both ciliated and microvillous OSNs in zebrafish [127]. Uranium (U) is a radioelement that can be found in contaminated water ecosystems both as its radioactive or depleted form. Waterborne U accumulates with great affinity in both the olfactory rosettes and olfactory bulbs, producing significant tissue damage to both structures. In the olfactory epithelium, the whole rosette seems affected, with ciliated OSNs and non-sensory cells showing more vulnerability [128,129,130]. Zinc (Zn) is both a common pollutant and a well-known olfactory toxicant used as an ingredient in over-the-counter intranasal drugs [131,132]. In zebrafish, it produces substantial damage to the olfactory sensory epithelium with a marked decrease in ciliated OSNs and an associated reduction of detection of bile salts and amino acids, to a lesser extent. Additionally, the anosmia produced by Zn treatment induces a stress response accompanied by anxiety-like behaviors and a reduction in locomotor activity [19,88,133].

These studies clearly show that exposure to waterborne heavy metals produce significant damage of the olfactory organ leading to olfactory dysfunction in zebrafish, with bile salt-sensing ciliated OSNs presenting the most susceptibility to the effects of some of these neurotoxicant agents (Figure 3A, Table 1). The functional consequences of this metal-induced olfactory impairment are several alterations in odor-mediated behaviors critical for survival (Figure 3C) [19,88,115,122,133].

### 4.2. Effects of Diverse Toxicants and Physical Lesioning on the Zebrafish Olfactory Epithelium

In addition to the effects of well-known neurotoxicants such as heavy metals, the olfactory system can be damaged by exposure to numerous chemical compounds and endogenous toxins, as well as by physical injury. Both chemical and physical lesions to the olfactory organs can result in olfactory epithelial damage, which severely impairs olfactory function and modifies odorant-driven behaviors (Figure 3A,C, Table 1).

Pesticides are widespread agricultural and domestic pollutants that have been strongly associated with olfactory impairment in several fish species. Chlorpyrifos (CPF) is a commercially available organophosphate pesticide extensively used for controlling agriculture and household pests. Exposure to CPF downregulates several genes related to olfactory sensing as well as neuronal growth, repair, and regeneration in the olfactory system of zebrafish [135]. Rotenone is another common pesticide that inhibits the function of mitochondrial complex I and particularly targets dopaminergic neurons. Rotenone exposure impairs olfactory response to amino acids in zebrafish and is accompanied by a reduction in dopamine production in the brain [136]. While the effects of rotenone on the olfactory organ were not examined in this study, it is possible that the olfactory dysfunction observed may be associated with a reduction in dopaminergic neurons in the olfactory bulb.

Surfactants are extensively used chemicals found in detergents, emulsifiers, foaming agents, among other commercially available products. Surfactants are routinely discarded on land and water systems and some of them can be toxic and produce olfactory impairment in fish [6]. Both acute and chronic intranasal irrigation with the detergent Triton X-100 cause severe morphological damage to the olfactory rosettes, including a thinning of sensory and non-sensory regions, OSN death, fused lamellae, and inflammation [18]. This extensive damage results in impairment of olfactory-mediated responses to bile salts and amino acids, to a lesser extent, suggesting that ciliated OSNs present more vulnerability to this type of chemical lesion [20,21,22]. It has been hypothesized that detergents solubilize cell membranes and lead to the release of intracellular components to the extracellular space, and that this underlies the extensive cell loss and damage observed with this type of chemical damage to the olfactory rosette [18]. It is unclear why some OSN subtypes appear to be more sensitive to detergent exposure.

It has been reported that patients with chronic kidney disease present severe olfactory dysfunction, although it is not known which uremic toxins produce this effect [143]. Studies in zebrafish investigated the effects of urea on the olfactory organ and found that chronic urea exposure produces a thinning of the olfactory epithelium accompanied by a reduction in OSN density, with crypt OSNs being the most sensitive cell type, followed by microvillous OSNs. Interestingly, it was also found that urea produces an upregulation of the ciliated OSN signaling G-protein, Gαolf [138,139].

An experimental paradigm for physical lesioning of the olfactory rosettes is acute and chronic wax plug insertions in the nasal cavity. These interventions produce significant and progressive damage of the olfactory organ, OSN death, and inflammation [140]. Mechanical stress has been proposed as one of the possible mechanisms responsible for the effects observed with this type of progressive damage.

An important and consistent observation emerging from these studies is that ciliated OSNs, responsive to bile salts, present an increased susceptibility to a diverse variety of toxicants and chemicals. It has been proposed that microvillous OSNs, which respond to amino acids, may present protective mechanisms that render them more resistant to damage, in order to preferentially preserve food sensing and feeding behaviors. It has also been suggested that these neurons may be replaced faster or in greater numbers and, therefore, their numbers do not plunge following olfactory epithelium damage [19,22,124].

In sum, evidence of the deleterious effects observed in the olfactory organ of zebrafish due to chemical exposure and direct injury supports the use of zebrafish as a robust model for studying the effects of an increased variety of toxicants and direct lesion paradigms in olfactory function.

### 4.3. Effects of Olfactory Toxicants and Injury on the Olfactory Bulb of Zebrafish

The blood–brain barrier (BBB) is a selective semipermeable barrier composed of the microvasculature of the CNS, endothelial cells, astrocytes, and pericytes, that tightly regulates the passage of molecules from the blood to the cerebrospinal fluid (CSF) surrounding the brain and spinal cord. This barrier is critical for maintaining CNS homeostasis and for protection against toxicants and pathogens present in the blood, inflammation, and disease. However, it is well known that molecules administered to the olfactory epithelium can reach the brain, bypassing the BBB via the olfactory and trigeminal nerves [144,145]. In fact, a great deal of effort has been made to develop intranasally delivered therapeutics to reach the CNS, since most molecules are unable to cross the BBB [144,145]. An adverse outcome of the circumvention of the BBB via the short olfactory nerve is that harmful chemicals and toxicants in direct contact with the olfactory epithelium have the potential to reach the olfactory bulb and the CSF within minutes (Figure 3B) [146,147].

It is well documented that olfactory exposure to heavy metals promotes their localization in the olfactory bulb in several fish species. Cd, Ni, and Zn are transported from the OSNs through axonal transport, where they reach the olfactory bulb [148,149,150,151]. In zebrafish, U greatly accumulates both in the olfactory epithelium and in the olfactory bulb. In the bulb, U causes morphological damage and severely disrupts glomerular structure, as well as dysregulating several genes related to olfactory sensing [128,129,130]. Waterborne silver (Ag) accumulates in the olfactory bulbs and promotes oxidative stress and the expression of genes related to oxidative damage [134]. No accumulation of Ag in the olfactory organ was reported. It was described that several heavy metals accumulate with great affinity in the olfactory bulb. It has been proposed that the presence of metal-binding molecules that are highly abundant in the olfactory bulb could underlie the vulnerability of this region to metal ions [150].

One of the consequences of OSN death produced by damage to the olfactory organ is the loss of projections of afferent axons from the OSNs to the olfactory bulb, in a process known as deafferentation. Olfactory deafferentation is used as a paradigm to study the influence of olfactory information in the development and maintenance of the olfactory bulb. Since zebrafish offers an amenable model for achieving chemical and physical damage to the olfactory rosettes, it has been widely used to study the effects of the loss of afferent input to the olfactory bulb.

Damage to the olfactory organ caused by chronic Triton X-100 exposure produces notable deafferentation of the olfactory bulb, leading to a significant reduction in bulb size and afferent activity [20,42]. Medial and dorsal glomeruli morphology was severely affected or reduced altogether, whereas lateral glomeruli were more resistant, consistent with previous reports that suggest that Triton X-100 treatment affects preferentially ciliated OSNs [21,22]. Additionally, this chemical treatment leads to profound changes in mitral cell structure and dendritic complexity [32,137].

Deafferentation of the olfactory bulb by physical injury to the olfactory organ is a common method of examining afferent–target interactions [32]. Physical damage to the zebrafish rosettes has been achieved experimentally by removing the rosettes entirely using a cautery iron or by progressively damaging the organ with a wax plug. Complete removal of the olfactory organ by cautery produces irreversible and complete deafferentation to the olfactory bulb, followed by severe bulb alterations. These include a significantly reduced bulbar size and activity; vast apoptosis; a reduction in survival and differentiation of newly generated bulbar neurons; complete degeneration of the olfactory nerve and the glomerular layer; and reduction in mitral cell dendritic complexity [32,41,42,137,141,142]. The olfactory bulbs of fish that receive nasal plug insertions show extensive deafferentation along with defasciculation and disruption of several glomeruli of the ventromedial cluster [140].

Overall, the results from these studies demonstrate that nasal exposure to heavy metals and toxicants, as well as physical damage to the olfactory organ, can lead to profound alterations in the olfactory bulb via axonal transport or deafferentation mechanisms (Figure 3B, Table 1). These results shed light on the mechanisms by which sensory activity conveyed by the olfactory organ promotes homeostasis and maintenance of the olfactory bulb.

### 4.4. Regeneration and Repair of the Zebrafish Olfactory System Following Damage

One outstanding feature of the zebrafish olfactory system is its extensive ability to regenerate and repair lesions swiftly following injury. Both the olfactory organ and the olfactory bulb present extensive remodeling and repair mechanisms as well as continuous generation of new OSNs and bulbar neurons throughout the organism lifespan [24,38,39,40,41,42]. These remarkable characteristics make the olfactory system of zebrafish an ideal model for studying regeneration, repair, and neurogenic mechanisms following damage and for understanding the role of innervation and reafferentation of the adult olfactory bulb as well.

The olfactory rosettes show bimodal patterns of OSN recovery following Cu exposure. On one hand, acute Cu exposure targets ciliated OSNs, with an observed recovery of this cell population after 72 h, whereas chronic exposure produces both ciliated and microvillous OSN loss with a faster proliferation and regeneration of microvillous OSNs observed after 72 h. Olfactory-mediated behavior assessment showed a partial functional recovery of ciliated OSNs, suggesting that although ciliated OSNs are more susceptible to Cu exposure, they are able to recover faster, perhaps through repair mechanisms rather than neurogenesis, a mechanism that was observed for the replenishment of microvillous OSNs [124].

Furthermore, both the olfactory organ and olfactory bulb can rapidly and completely recover to control morphology and activity following different types of damage to the olfactory epithelium: (1) acute Triton X-100 exposure; (2) chronic Triton X-100 exposure; and (3) progressive physical injury with wax plug insertions (Figure 3A,B, Table 1). Following acute exposure to Triton X-100, the olfactory epithelium is regenerated in 5 days, and reinnervation to the olfactory bulb is achieved at 7 days; additionally, at this time point, olfactory bulb volume is restored and the vast majority of affected glomeruli recover their size, shape, and specific location within the bulb. Glomeruli of the medial and dorsal clusters show partial recovery. After 10 days, lesioned fish regain an olfactory response to bile salts, showing a functional correlate of the glomerular reinnervation pattern observed and suggesting that, although glomerular structure recovers by 7 days, synaptic connections with dendrites of bulbar neurons require additional time to take place [18,21,22,32]. On the other hand, when the olfactory organ is chronically lesioned with Triton X-100, recovery of both olfactory organ structure and olfactory bulb morphology, volume, and afferent activity is observed after 21 days [20]. Moreover, following physical lesioning with nasal plug insertions, the olfactory epithelium returns to control morphology, size, and volume within 7 days. At this time point, partial innervation to the olfactory bulb is observed, as well as reformation of discrete glomeruli. By 21 days, complete bulbar innervation as well as glomerular morphology and distribution are achieved [140].

The remarkable ability of the adult zebrafish olfactory organ to regenerate following different types of damage and lesioning can be explained since the olfactory epithelium presents continuous neurogenesis, due to the presence of neuronal precursors in the basal cell layer of the epithelium. In control conditions, OSNs present a constant turnover rate and most proliferation occurs in discrete clusters located in the apex of the lamellae [13,38,40,152]. Following damage to the olfactory organ, cellular proliferation is increased and localized in broader regions on the olfactory epithelium, giving rise to a rapid recovery of olfactory organ size and function [18,123,124]. Newly generated OSNs can reinnervate the olfactory bulb in their corresponding glomeruli and make synaptic connections with mitral cells. Following partial or complete damage of the olfactory organ, this process occurs in a rapid and coordinated fashion, allowing a complete recovery of bulbar morphology and function within weeks. It has been proposed that bulbar reafferentation by OSNs is one of the early mechanisms that promote complete recovery of the olfactory bulbs following damage to the olfactory epithelium. Studies on the complete ablation of the olfactory organ by means of cautery support this idea, since irreversibly deafferentated olfactory bulbs do not recover their size, morphology, or structure following OSN ablation [32,140,141,142].

Furthermore, it was shown that there is an increase in cellular proliferation in the olfactory bulbs and adjacent ventricular zone following an olfactory organ lesion, and that non-neuronal newly born cells likely contribute to the recovery of olfactory bulb volume. On the other hand, most newly born neurons generated following OSN damage do not survive nor integrate into bulbar circuits. It has been shown in mammals that the survival and integration of newly generated neurons in the olfactory bulb requires adequate sensory input during a restricted time window [153,154]. Thus, it is possible that most newborn neurons generated in the deafferentated bulb of zebrafish do not survive because they are unable to form strong synaptic connections with OSN axons [39,41,42].

Cumulatively, these studies show that both the olfactory organ and olfactory bulb of zebrafish show extensive regeneration and recovery abilities following peripheral damage with a variety of injury paradigms (Table 1). Moreover, they report that the time course of regeneration and repair of the lesioned olfactory system of zebrafish is much faster than what is observed in other vertebrates, a feature that makes this model attractive for regeneration studies since it allows for faster recovery times [155,156]. These results also support the idea that ongoing and diverse plasticity mechanisms in the olfactory system underlie optimal olfactory function, suggesting that there is not a critical period after which the olfactory bulb becomes unresponsive to afferent stimuli [157,158,159,160].

## 5. Conclusions

Given the widespread occurrence of airborne and waterborne olfactory toxicants such as heavy metals, pesticides, surfactants and general pollutants, there is a pressing need to understand the effects of these harmful compounds on olfactory function. The studies reviewed here shed light on the anatomical, morphological and functional architecture of the zebrafish olfactory system, as well as describing the impact of chemical and physical injury on olfactory function. Furthermore, the regeneration and repair abilities of the zebrafish olfactory system allow for the study of regeneration mechanisms absent or reduced in mammals. Studies reviewed here improve our understanding of diverse fields, including molecular basis of olfaction and olfactory-mediated behavior, olfactory toxicity, neuroplasticity, regeneration mechanisms following damage, among others. These will also provide a foundation for further studies on these topics, with several implications for understanding human health and disease. In conclusion, the cumulative evidence presented here validates the use of zebrafish as a robust model for studying the molecular basis of olfaction, as well as the consequences of toxicant exposure and physical damage to the olfactory organ.

## Figures and Tables

**Table 1:** Effects of various olfactory toxicants and injury paradigms on the olfactory epithelium (OE), olfactory bulb (OB), and on olfactory-mediated behaviors of zebrafish, as well as their recovery.

Toxicant or Injury Paradigm	Effects on OE	Effects on OB	Behavioral Effects	Recovery	Ref.
Cadmium (Cd)	OSN loss, reduced neurogenesis, ROS increase		Long-term decrease in alarm and avoidance responses		[88,114,115,116,117,118,119]
Copper (Cu)	Predominantly ciliated OSN loss, decreased OR and ionic channels transcripts		Impaired response to bile salts, reduced alarm response	OSN recovery after 72 h. Partial recovery of bile salt response.	[120,121,122,123,124]
Cobalt (Co)	Acute damage to OE, apoptosis and increased ROS		Alterations in schooling behavior		[125,126]
Nickel (Ni)	OSN loss and OO anatomical disturbances				[127]
Uranium (U)	Ciliated OSNs and non-sensory cells damage and loss	Morphological damage and disruption of glomerular structure			[128,129,130]
Zinc (Zn)	Damage to the sensory epithelium with ciliated OSN loss		Impaired response predominantly to bile salts, and aminoacids. Anxiety-like behaviors and reduction in locomotion		[19,88,133]
Silver (Ag)		Oxidative stress and expression of oxidative damage genes			[134]
Chlorpyrifos	Decrease in transcripts related to olfactory sensing and neuronal repair and regeneration				[135]
Rotenone			Impaired response to amino acids		[136]
Triton X-100	Thinning of OE, fused lamellae, inflammation and OSN loss	Deafferentation, glomerular defasciculation, reduction of size and activity. Mitral cell structural alterations	Reduced response to predominantly bile salts, and aminoacids	Following acute exposure, OE regenerates in 5 days; bulbar reinnervation observed in 7 days; functional olfactory recovery at 10 days. Following chronic exposure, OE and OB structure, activity and volume are recovered at 21 days.	[18,20,21,22,32,42,137]
Urea	Thinning of OE with crypt OSN loss, upregulation of G_α_olf transcript				[138,139]
Chronic physical olfactory organ lesion	OE damage, inflammation and OSN loss	Bulbar deafferentation and glomerular defasciculation		OE regeneration and OB recovery observed in 7 days. Complete bulbar reinnervation at 21 days	[140]
Olfactory organ removal		Reduction in size and activity. Complete deafferentation, degeneration of both olfactory nerve and glomerular layer. Increased apopotsis			[32,41,42,137,141,142]
